# Bloodstream infections epidemiology and clinical outcomes after 1 year post allogeneic hematogenous stem cell transplantation

**DOI:** 10.3389/fmicb.2025.1596900

**Published:** 2025-08-07

**Authors:** Silvia Corcione, Bianca Maria Longo, Walter Rugge, Ilaria De Benedetto, Tommaso Lupia, Nour Shbaklo, Sofia Zompi, Jessica Gill, Antonio Curtoni, Roberto Passera, Alessandro Busca, Benedetto Bruno, Francesco Giuseppe De Rosa

**Affiliations:** ^1^Department of Medical Sciences, University of Torino, Torino, Italy; ^2^School of Medicine, Tufts University, Boston, MA, United States; ^3^Unit of Infectious Diseases, Cardinal Massaia, Asti, Italy; ^4^Unit of Infectious Diseases, Department of Medical Sciences, AOU Città della Salute e della Scienza di Torino, University of Turin, Torino, Italy; ^5^Department of Oncology, Stem Cell Transplant Center, Città della Salute e della Scienza Hospital, Turin, Italy; ^6^Department of Molecular Biotechnologies and Health Sciences, University of Torino, Torino, Italy; ^7^Microbiology and Virology Unit, University Hospital Città della Salute e della Scienza di Torino, Torino, Italy; ^8^Division of Hematology, AOU “Città della Salute e della Scienza di Torino”, Torino, Italy

**Keywords:** HSCT, bloodstream infections, MDR, hematology, colonization

## Abstract

**Background:**

Bloodstream infections (BSIs) are a serious threat for patients undergoing allogenic hematopoietic stem cell transplants (a-HSCT). MDR colonization is highly prevalent among a-HSCT patients, due to drug-induced intestinal dysbiosis. Primary outcome of the study was to assess the epidemiology and risk factors for BSIs in the 1st year after a-HSCT. Secondary endpoints were to examine the prevalence of MDR bacterial colonization and factors affecting 1-year post-transplant overall survival (OS).

**Methods:**

In this single-center observational cohort study, all consecutive adult patients undergoing a-HSCT for hematological malignancies between 2012 and 2021 were retrospectively enrolled at the Stem Cell Transplant Center, AOU Città della Salute e della Scienza, Turin (Italy). Cumulative Incidence and risk factors for BSIs were analyzed by Gray and Fine-Gray tests, respectively. OS was evaluated with Kaplan–Meier, while the influence of covariates on OS with Cox regression analysis.

**Results:**

Two hundred and seventy nine patients were enrolled in the study, 43% of which developed BSI within the 1st-year post-transplant. The median onset of BSIs was 10 days after a-HSCT and Gram-negative bacteria were the most common causative agents (58.3%). 20.8% of patients had a positive rectal swab (RS) for MDR bacteria, with extended-spectrum β-lactamases (ESBLs)-producing *Enterobacterales* being the most common colonizers (60.3% of positive RS), followed by carbapenem-resistant Enterobacteriaceae (CRE) (29.3%). Multivariate competing risks regression analysis showed that colonization by MDR bacteria was associated with a higher incidence of BSIs (SDHR 1.49, 95%CI 1.01–2.20), along with the type of underlying disease (SDHR 0.73, 95%CI 0.58–0.91), donor type (SDHR 1.62, 95%CI 1.02–2.58) and an advanced disease status at the time of transplantation (SDHR 1.57, 95%CI 1.05–2.35). One-year mortality rate was 25.4%. RS colonization was not associated with increased mortality; while BSIs adversely affected OS (SDHR 1.52, 95%CI 1.02–2.26).

**Conclusions:**

BSIs are common complications in a-HSCT, with evidence suggesting a negative impact on OS. Although MDR colonization is not independently linked to increased mortality in a-HSCT, it appears to be associated with an elevated risk of subsequent BSI development. These findings underscore the potential value of pre-transplant surveillance, contact precautions, and early targeted antimicrobial therapy in colonized patients to help mitigate infection-related morbidity and mortality.

## 1 Introduction

More than 50,000 hematopoietic stem cell transplantations (HSCTs) are performed every year all over the world, both for malignant and non-malignant hematological diseases (Henig and Zuckerman, 2014). In allogeneic HSCT (a-HSCT), donor stem cells, harvested from another individual or from umbilical cord blood units, are used to replace recipient stem cells, previously destroyed by conditioning regimens (Ringdén et al., 2009). Exposure to conditioning regimens themselves, myeloablation and immunosuppressive agents along with the occurrence of acute/chronic graft vs. host disease (GvHD) are some of the factors predisposing a-HSCT patients to develop serious, often life-threatening, infections (Sahin et al., 2016).

In the broad spectrum of infections that patients may develop following a-HSCT, bloodstream infections (BSIs) are a major threat, especially in the pre-engraftment phase (Mikulska et al., 2009). Historically, the mainly involved pathogens were Gram-positive bacteria with a rising rate of resistance to penicillins and fluoroquinolones (Collin et al., 2001). Epidemiology of BSIs in a-HSCT has recently changed with a remarkable increase in Gram-negative bacilli (Macesic et al., 2014) and the problem of multidrug-resistant (MDR) bacteria is an issue that clinicians face in daily clinical practice (Sievert et al., 2013). In recent decades, there has been a significant rise in BSIs caused by Extended Spectrum Beta-lactamases (ESBL)-producing and carbapenem-resistant Enterobacteriaceae (CRE). This increase parallels the prevalence of MDR *Pseudomonas aeruginosa* and, among Gram-positive bacteria, vancomycin-resistant Enterococci (VRE) (Satlin et al., 2014; Satlin and Walsh, 2017).

Overall, BSIs have been demonstrated to exert an unfavorable prognostic impact on the outcome of hematological patients undergoing a-HSCT (Trecarichi et al., 2023; Girmenia et al., 2017). This seems to be especially true in the case of infections sustained by MDR pathogens (Girmenia et al., 2017; Trecarichi et al., 2019, 2016). In a large multicenter study conducted in 15 Italian hematological wards, resistance to 3rd generation cephalosporins among *Escherichia coli*, identified in the 25.7% of the isolates, was a significant 30-day mortality predictor, together with acute hepatic failure, septic shock, male sex, refractory/relapsed malignancy (Trecarichi et al., 2019). Moreover, resistance to carbapenems dramatically increases the overall mortality among patients suffering from hematological malignancies with BSIs caused by *Klebsiella pneumoniae* as described by (Trecarichi et al. 2016), and inadequate initial therapy is another crucial risk factor for increased mortality.

Since MDR colonization is a recognized risk factor for development of MDR-BSI in the general population (Giannella et al., 2014), some studies have started to focus on epidemiology and implications of MDR bacterial colonization among hematological patients (Henig and Zuckerman, 2014; Cattaneo et al., 2018). Indeed, patients undergoing a-HSCT are particularly prone to acquiring MDR rectal colonization due to infection control measures, local epidemiology, intestinal dysbiosis favored by chemotherapy and extensive antibiotic use (Chong et al., 2017). The emergence of MDR bacteria in the setting of a-HSCT recipients, may raise critical concerns about transplant eligibility of colonized patients and potential for infection outbreaks within the transplant center. In this scenario, the relationship between colonization and the development of infections, particularly BSIs, has also been investigated (Trecarichi et al., 2023; De Rosa et al., 2017).

The aim of this study was to analyze epidemiology and risk factors for BSIs during the 1st-year post-a-HSCT. We also aimed to describe the epidemiology of MDR colonization in a-HSCT patients and to identify factors affecting overall survival (OS) within the first year after a-HSCT.

## 2 Materials and methods

### 2.1 Study design

We performed a single-center observational retrospective cohort study including all consecutive adults (≥18-year-old) undergoing a-HSCT for hematological malignancy between August 2012 and October 2021 at the Stem Cell Transplant Center, AOU Città della Salute e della Scienza, Turin (Italy).

The primary endpoint was to report the epidemiology and risk factors for BSI occurring in the 1st year post-HSCT. Secondary endpoints were to describe the epidemiology of MDR-bacteria intestinal colonization during hospitalization for a-HSCT and its relationship with the development of BSI and defining risk factors for the OS during the 1st year after a-HSCT.

The study was conducted according to the principles of the Declaration of Helsinki and the Declaration of Istanbul and was approved by our Institutional Ethics Committee (resolution no. 506/2021). All the participants signed a written consent for transplant procedure as previewed by clinical practice and for the retrospective use of anonymous medical data for research purpose.

### 2.2 Demographic and HSCT data

Demographic and clinical data were retrieved through the revision of a computerized database of prospectively collected data, updated until October 2023. Indication for a-HSCT were hematologic malignancies including Acute Myeloid Leukemia (AML), Myelodysplastic syndromes (MDS), Acute Lymphoblastic Leukemia (ALL), Lymphomas, Chronic Myeloid Leukemia (CML) and Multiple Myeloma (MM). We excluded transplants performed for non-hematologic malignancies or for solid tumors. Patients receiving multiple a-HSCT during the study period were censored at the time of second or third a-HSCT and each single transplant was analyzed independently from the others. Both acute-Graft-vs.-host disease (a-GVHD) and chronic-Graft-vs.-host disease (c-GVHD) were diagnosed based on clinical symptoms and/or biopsies according to standard criteria (Malard et al., 2023; Shulman et al., 2015). Neutropenia was defined as an absolute neutrophil count < 500/mm^3^. Transplant risk for each patient was calculated using the Hematopoietic Cell Transplantation-Specific Comorbidity Index (HSCT-CI) (Sorror et al., 2005). Patients with febrile neutropenia were treated in accordance with internal protocols (i.e., piperacillin/tazobactam or cefepime). In instances of fever or febrile neutropenia without septic shock, an additional drug with possible KPC action, such as tigecycline and/or an aminoglycoside, was included into the empirical antibiotic regimen for febrile neutropenia. In the event of septic shock or clinical deterioration, an empirical therapy incorporating an anti-KPC agent (e.g., ceftazidime/avibactam, meropenem/vaborbactam, imipenem/cilastatin/relebactam, cefiderocol) was included into the therapeutic regimen. Moreover, patients at risk with confirmed rectal ESBL presenting with fever or febrile neutropenia, without septic shock, had tigecycline and/or an aminoglycoside included into their empirical antibiotic regimen. In the event of septic shock or clinical deterioration, an empirical therapy including a high-dose carbapenem and aminoglycosides was incorporated into the therapeutic regimen. Moreover, patient care was provided adhering to infection prevention and control standards, including proper use of PPE, contact precautions, barrier nursing, and environmental cleaning.

### 2.3 Bloodstream infections definitions and diagnostic technique

BSI was defined as the isolation of one or more bacterial or fungal pathogens in a blood culture, excluding organisms listed as common commensals by the NHSN (Centers for Disease Control and Prevention, 2025). Coagulase-negative staphylococci (CoNS) and other typical skin contaminants were considered significant if the patient showed clinical signs of infection—such as fever, low blood pressure, chills, or rigors—and the same organism was isolated in at least two separate blood cultures. BSIs were defined as “polymicrobial” if more than one bacterial microorganism was detected on the 1st day of a BSI episode. Only BSI occurring in the 1st year from transplantation were considered and, in case of multiple and relapsing bacterial BSI, only the first episode was included in the analysis. Incidence data were based on the rate of BSI per the yearly number of transplants. Cultures were analyzed with the BD BACTECTM FX system (Becton Dickinson) according to the European Committee on Antimicrobial Susceptibility Testing (EUCAST) breakpoint tables (European Committee on Antimicrobial Susceptibility Testing, 2025). The identification of microorganisms was conducted with matrix-assisted laser desorption ionization time-of-flight mass spectrome- try and VITEK^®^, whereas susceptibility to antibiotic molecules was tested using VITEK 2 (VITEK^®^ according to EUCAST breakpoint tables).

### 2.4 MDR colonization sampling

Patients were screened with a rectal swab to detect ESBL-producers and CR Enterobacteriaceae, as well as MDR non-fermenting GNB. Carbapenemases have been considered as KPC, the most common carbapenemase based on local epidemiology, unless otherwise specified. Biomolecular test for detection of ESBL strains on RS was introduced in our center in 2017, so it was not possible to assess the prevalence of these rectal colonizers before 2017. Gram negative bacteria were considered MDR if they produced ESBL or carbapenemases, and in all cases of resistance to 1 agent in 3 therapeutically relevant classes of antibiotics. Our screening policy did not cover vancomycin-resistant Enterococcus faecium colonization. Screening was performed from the admission for transplant induction and then weekly until discharge.

### 2.5 Statistical analysis

Primary endpoint was the cumulative incidence (CI) for BSI at 1–3–6–12 months from the day of a-HSCT (main event); competing event was the relapse/death without BSI, alive patients were censored at the date of last contact. The CI function was compared across groups by the Gray-test, while the competing risks regression model was used to estimate the role of risk factors on BSI occurrence by the Fine-Gray test. The following covariates were selected by investigator consensus and tested as BSI occurrence determinants: recipient age (>60 vs. 40–60 vs. < 40 years), recipient gender (female vs. male), diagnosis (AML/MDS vs. ALL vs. other hematological malignancies), disease status at transplant (advanced vs. early disease), Hematopoietic cell transplantation comorbidity index (HCT-CI, ≥3 vs. 0–2), donor type (haploidentical vs. matched unrelated donor vs. matched related donor), stem cell source (peripheral blood vs. bone marrow), conditioning regimen (reduced intensity vs. myeloablative), anti-thymocyte globulin (ATG) administration as GvHD prophylaxis (yes vs. no), antibiotic prophylaxis (yes vs. no), BSI occurrence (yes vs. no) and etiology, RS (positive vs. negative), acute (grade II–IV vs. 0–I), and chronic GVHD (moderate/severe vs. no/mild) occurrence. Secondary endpoint was OS, defined as the time from transplant to death from any cause. Survival curves were estimated by the Kaplan–Meier method and compared across groups by the log-rank test. The effect on OS of the same set of covariates was analyzed by the Cox proportional hazards regression model, comparing the two arms by the Wald test, and calculating 95% confidence intervals (95%CI). Patient characteristics were tested using Fisher's exact test for categorical variables and the Mann–Whitney and Kruskal–Wallis tests for continuous ones; continuous variables were described as median (Inter Quartile Range-IQR). All reported *p-*values were obtained by the two-sided exact method at the conventional 5% significance level. Data were analyzed as of October 2024 by R 4.4.2 (R Foundation for Statistical Computing, Vienna-A, http://www.R-project.org, accessed on November 21, 2024).

## 3 Results

### 3.1 Patients' characteristics

Two hundreds and seventy-nine patients undergoing a-HSCT were included in the study and clinical and microbiological characteristics were summarized in Table 1. A slight prevalence of male (*N* = 153, 54.8%) was found and median age was 52 years (range 18–69). AML/MDS were the most common malignancies (63.6%) leading to a-HSCT in our population. Most of the patients (82.1%) were in complete remission (CR) or partial remission (PR) at the time of transplantation, while 17.9% had an advanced stage of disease. More than a quarter of the patients had an HCT-CI above 3 (*N* = 82, 29.4%).

**Table 1:** Clinical and microbiological characteristics of the whole cohort and stratified by colonization status.

**Characteristics**	**All patients**	**Positive RS**	**Negative RS**	***p*-value**
**Sex**				
Male	153 (54.8%)	35 (22.9%)	118 (77.1%)	*p =* 0.37
Female	126 (45.2%)	23 (18.3%)	103 (81.7%)	
**Age (years)**				
< 40	62 (22.2%)	17 (27.4%)	45 (72.6%)	*p =* 0.31
41–60	138 (49.5%)	25 (18.1%)	113 (81.9%)	
>61	79 (28.3%)	16 (20.3%)	63 (79.7%)	
**Diagnosis**				
AML/MDS	176 (63%)	24 (19.3%)	142 (80.7%)	*p =* 0.53
ALL	45 (16.1%)	12 (26.7%)	33 (73.3%)	
Other	58 (20.8%)	12 (20.7%)	46 (79.3%)	
**Disease status**				
early	229 (82.1%)	43 (18.8%)	186 (81.2%)	*p =* 0.085
advanced	50 (17.9%)	15 (30.0%)	35 (70%)	
**HCT-CI**				
0	79 (28.3%)	23 (29.1%)	56 (70.9%)	*p =* 0.005
1-2	118 (42.3%)	14 (11.9%)	104 (88.1%)	
3+	82 (29.4%)	61 (74.4%)	21 (25.6%)	
**Donor type**				
MRD	67 (24.0%)	12 (17.9%)	55 (82.1%)	*p =* 0.29
Haploidentical	56 (20.1%)	16 (28.6%)	40 (71.4%)	
MUD	156 (55.9%)	30 (19.2%)	126 (80.8%)	
**HSC source**				
PBSC	223 (83.5%)	49 (21.0%)	184 (79%)	*p =* 1
BM	46 (16.5%)	9 (19.6%)	37 (80.4%)	
**Conditioning**				
MAC	230 (82.4%)	46 (20.0%)	184 (80.0%)	*p =* 0.56
RIC	49 (17.6%)	12 (24.5%)	37 (75.5%)	
**ATG**				
No	126 (45.2%)	29 (23.0%)	97 (77.0%)	*p =* 0.45
Yes	153 (54.8%)	29 (19.0%)	124 (81.0%)	
**aGVHD**				
No	127 (45.5%)	24 (28.9%)	103 (81.1%)	*p =* 0.55
Yes	152 (54.5%)	34 (22.4%)	118 (77.6%)	
**cGVHD**				
No	175 (69.5%)	37 (21.1%)	138 (78.9%)	*p =* 0.88
Yes	104 (37.3%)	21 (20.2%)	83 (79.8%)	
**ATB Prophylaxis**				
No	194 (69.5%)	52 (26.8%)	142 (73.2%)	*p < * 0.001
Yes	85 (30.9%)	6 (7.1%)	79 (92.9%)	
**BSI**				
No	159 (57%)	27 (17.0%)	132 (83.0%)	*p =* 0.76
Yes	120 (43%)	31 (25.8%)	89 (74.2%)	
**RS**				
Neg	221 (79.2%)	–	–	–
Pos	58 (20.8%)			

RS, rectal swab; a-HSCT, allogenic-haematopoietic stem cell transplantation; AML, acute myeloid leukemia; MDS, myelodysplastic syndrome; ALL, acute lymphoblastic leukemia; CR, complete response; PR, partial response; SD, stable disease; PD, progression disease; HCT-CI, hematopoietic cell transplantation comorbidity index; MRD, matched related donor; MUD, matched unrelated donor; HSC, haematopoietic stem cell; PBSC, peripheric blood stem cell; BM, bone marrow; MAC, myeloablative conditioning; RIC, reduced intensity conditioning; ATG, anti-thymocytes globulin; aGVHD, acute graft vs. host disease; cGVHD, chronic graft vs. host disease; ATB prophylaxis, antibiotic prophylaxis; BSI, blood stream infection. For both the “positive RS” and “negative RS” groups, the percentage was calculated based on the total number of patients (column 2) stratified by each variable (e.g., sex, age, etc).

Overall 67 patients (24.0%) received a-HSCT from a matched related donor, (MRD) while 212 patients (76%) were grafted from alternative donors, including a matched unrelated donor (MUD) in 156 cases and haploidentical donors in 56 cases. Myeloablative conditioning (MAC) regimen was used in 82.4% of the transplants, while for the remainder (17.6%) a reduced-intensity conditioning (RIC) was preferred. Peripheral blood was the graft source in 83.5% of the patients. GVHD prophylaxis included ATG in 54.8% of the cases. Overall, 85 patients (31%) received antibacterial prophylaxis with levofloxacin (most of them were grafted before 2017). Acute-GVHD occurred in 54.5% of patients, while cGVHD was reported in 37.3% of the patients.

### 3.2. Epidemiology of BSIs

Overall, 120 patients (43%) developed a BSI during hospitalization. The CI of BSI at 1, 3, 6, and 12 months after a-HSCT was 30.8%, 37.3%, 39.1%, and 43.0%, respectively. In Table 2 was reported CI of bacterial BSI following a-HSCT according to study population characteristics.

**Table 2:** Bloodstream infections cumulative incidence according to clinical and microbiological factors.

	**BSI cumulative incidence at 1-3-6-12 months after a-HSCT (%)**	**Gray test *p*-value**
Overall, whole cohort	30.8–37.3–39.1–43.0	–
**Rectal swab**		
Negative	28.5–34.4–36.2–40.3	0.052
Positive	41.4–48.3–50.0–53.4	
**Age (years)**		
≤ 40	35.5–38.7–40.3–40.3	0.117
41–60	31.9–42.0–42.8–49.3	
61+	25.3–27.8–31.6–34.2	
**Gender**		
Male	32.7–37.2–39.2–44.4	0.593
Female	28.6–37.3–38.9–41.3	
**Underlying disease**		
AML/MDS	34.1–40.3–42.0–46.6	0.01
ALL	37.8–44.4–46.7–51.1	
other	15.5–22.4–24.1–25.9	
**Disease status at transplant**		
Early	30.1–35.4–37.1–40.6	0.065
Advanced	34.0–46.0–48.0–54.0	
**HCT-CI**		
0–2	31.6–34.2–38.0–44.3	0.798
≥3	28.8–38.1–38.1–40.7	
**Donor type**		
MRD	16.4–19.4–22.4–25.4	0.004
Haploidentical	35.7–42.9–44.6–48.2	
MUD	35.3–42.9–44.2–48.7	
**HSC source**		
PBSC	31.8–38.2–39.5–42.9	0.893
BM	26.1–32.6–37.0–43.5	
**ATG administration**		
No	24.6–30.2–32.5–35.7	0.031
Yes	35.9–43.3–44.4–49.0	
**Conditioning regimen**		
MAC	28.7–35.7–37.0–41.3	0.127
RIC	40.8–44.9–49.0–51.0	
**ATB prophylaxis**		
No	34.0–40.2–42.3–45.9	0.128
Yes	23.5–30.6–31.8–36.5	
**aGvHD**		
0–I	31.5–37.0–38.6–42.5	0.998
II–IV	30.3–37.5–39.5–43.4	

BSI, bloodstream infections; a-HSCT, allogenic haematopoietic stem cell transplantation; AML, acute Abbreviations, BSI, bloodstream infections; a-HSCT, allogenic haematopoietic stem cell transplantation; AML, acute myeloid leukemia; MDS, myelodysplastic syndrome; ALL, acute lymphoblastic leukemia; CR, complete response; PR, partial response; SD, stable disease; PD, progression disease; HCT-CI, hematopoietic cell transplantation comorbidity index; MRD, matched related donor; MUD, matched unrelated donor; HSC, haematopoieti stem cell; PBSC, peripheric blood stem cell; BM, bone marrow; ATG, anti-thymocytes globulin; MAC, myeloablative conditioning; RIC, reduced intensity conditioning; ATB prophylaxis, antibiotic prophylaxis; aGVHD, acute graft vs. host disease

Gram-negative bacteria were responsible for BSI in the majority of cases (58.3%). Among these, 58 (48.3%) were Enterobacteriaceae, with *E. coli* and *K. pneumoniae* being the most frequently isolated. Conversely, non-fermenting Gram-negative bacteria accounted for 10% of cases, with *P. aeruginosa* being the predominant species within this group. Gram-positive bacteria were isolated in 41.7% (50 cases) of instances, with methicillin-resistant *S. epidermidis* (MRSE) and other coagulase-negative staphylococci being the most prevalent (refer to Table 3).

**Table 3:** Bloodstream infections and rectal swab microbiology.

**BSI etiology**	**BSI (*N =* 120)**
Enterobacteriaceae	58 (48.3%)
Non-fermenting Gram-negative bacteria	12 (10%)
Gram-positive bacteria	50 (41.7%)
**Rectal swab microbiology**	**Positive swabs (*****N** =* **58)**
ESBL-Enterobacteriaeae	35 (60.3%)
CRE	17 (29.3%)
CR-PA	6 (10.3%)

BSI, bloodstream infections; ESBL, extended spectrum β-lactamase; CRE, carbapenem-resistant Enterobacteriaceae; CR-PA, carbapenem-resistant Pseudomonas aeruginosa.

### 3.3 Epidemiology of MDR bacterial colonization

MDR bacterial colonization screening was conducted on the entire population using RS. Of the 58 positive swabs (20.8%), 32 (55.2%) were collected on pre-transplant days and 26 (44.8%) after transplant. As shown in Table 3, ESBLs-producing Enterobacteriaceae were confirmed to be common colonizers (60.3% of positive swabs), followed by carbapenem-resistant Enterobacteriaceae (CRE) (29.3%). Only 6 patients were colonized by carbapenems-resistant *P. aeruginosa* (CRPA). Table 1 shows that the colonization rate in age groups up to 40, 41–60 and 61 years and older were 27.4%, 18.1%, and 20.3%, respectively. When stratifying colonization prevalence based on the underlying hematological condition, RS positivity was found in 26.7% of patients with ALL and 19.3% of those with AML/MDS. Although not statistically significant, a higher colonization rate (30% vs. 18.8%, *p* = 0.085) was observed in patients with advanced disease compared to those in CR/PR. Additionally, a greater colonization rate was noted in patients with a higher HCT-CI (≥3) (74.4%, vs. 25.6% *p* = 0.005). Only 7.1% of patients on fluoroquinolone prophylaxis were colonized, compared to 26.8% of patients who did not take FQ prophylaxis.

### 3.4 Risk factors for BSI

In Table 4, we reported the univariate and multivariate competing risks regression analyses for bacterial BSI after a-HSCT. A positive RS (SDHR 1.49, 95%CI 1.01–2.20), underlying disease (other vs. ALL vs. AML/MDS, SDHR 0.73, 95%CI 0.58–0.91), donor type (MUD vs. haploidentical vs. MRD, SDHR 1.62, 95%CI 1.02–2.58) and an advanced disease status at time of transplant (advanced vs. early, SDHR 1.57, 95%CI 1.05–2.35), were associated with a higher CI of bacterial BSIs at multivariate analysis.

**Table 4:** Univariate and multivariate competing risk regression for BSI 1 year after a-HSCT.

	**Univariate models**		**Multivariate model**	
	**SDHR(95% CI)**	**Fine-gray test** ***p*****-value**	**SDHR (95% CI)**	**Fine-gray test** ***p*****-value**
Rectal swab (positive vs. negative)	1.49 (1.00–2.21)	0.050	1.49 (1.01–2.20)	0.045
Underlying disease (other vs. ALL vs. AML/MDS)	0.75 (0.60–0.94)	0.012	0.73 (0.58–0.91)	0.005
Disease status at transplant (advanced vs. early)	1.48 (0.98–2.25)	0.062	1.57 (1.05–2.35)	0.027
Donor type (MUD vs. Haploidentical vs. MRD)	1.39 (1.12–1.73)	0.003	1.62 (1.02–2.58)	0.040
ATG administration (yes vs. no)	1.48 (1.03–2.12)	0.032	0.81 (0.40–1.64)	0.550

SDHR, sub-distribution hazard ratio; AML, acute myeloid leukemia; MDS, myelodysplastic syndrome; ALL, acute lymphoblastic leukemia; ALL, acute lymphoblastic leukemia; MUD, matched unrelated donor; MRD, matched related donor; ATG, anti-thymocytes globulin.

**Figure 1:**
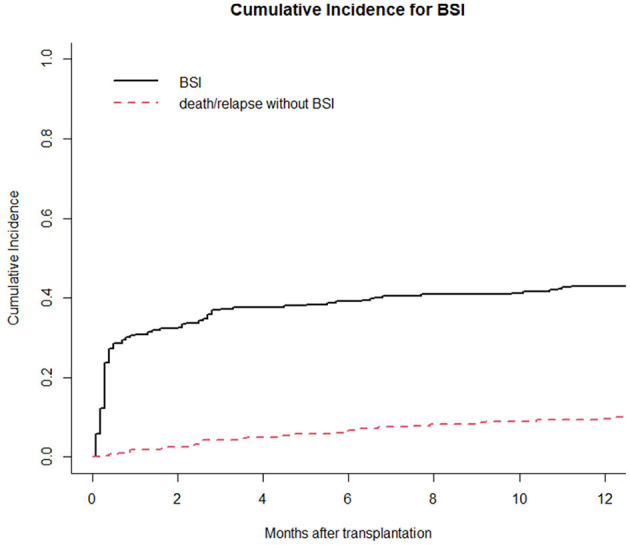
Cumulative incidence of bacterial BSI in the 1st year after a-HSCT. Death/relapse without BSIs is considered the competing event.

**Figure 2:**
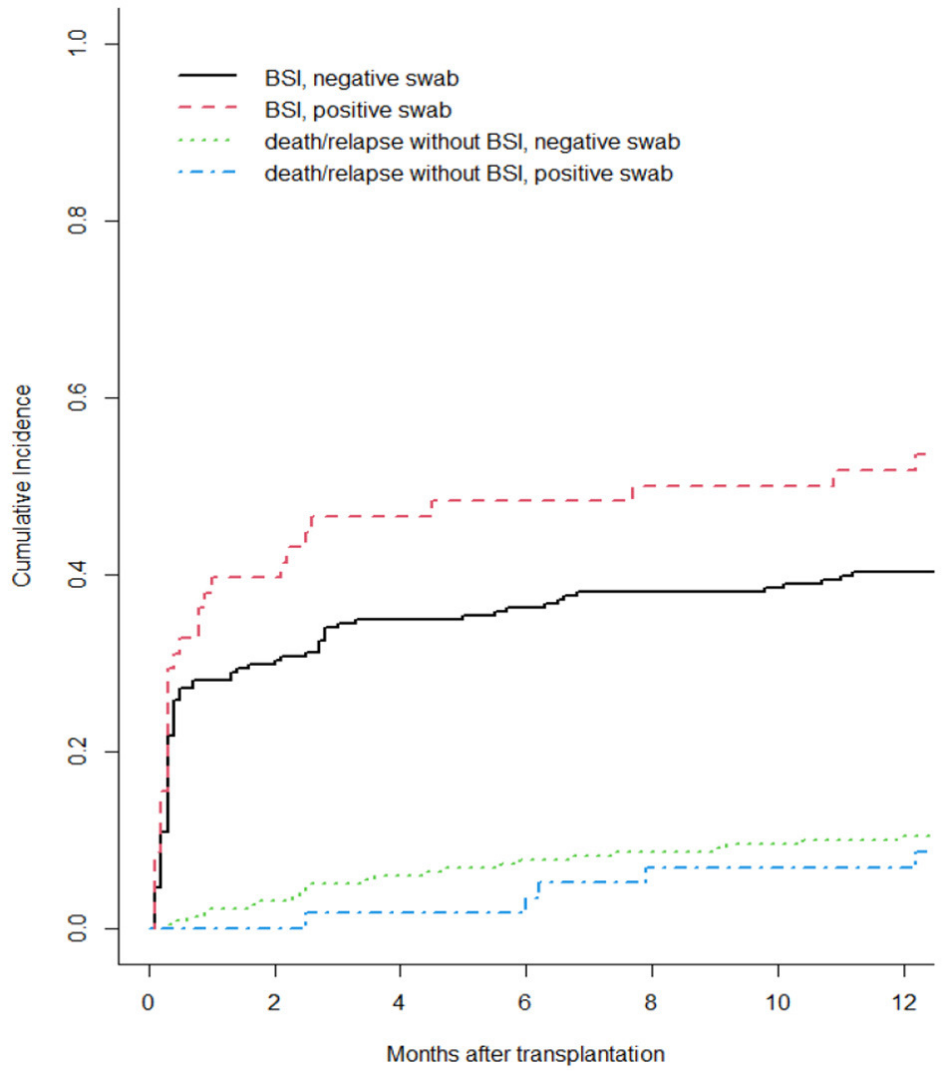
Cumulative incidence of bacterial BSI after a-HSCT in the 1st year after a-HSCT, stratified by colonization status. Death/relapse without BSIs were considered the competing event.

Moreover, we reported in Figure 1 the CI of bacterial BSI after a-HSCT in the 12-months following transplant (CI BSI at 1/3/6/12 months: 30.8%/37.3%/39.1%/43.0%, while Figure 2 differentiates CI based on RS result (CI BSI with negative swab at 1/3/6/12 months: 28.1%/34.4%/36.2%/40.3%; CI BSI with positive swab at 1/3/6/12 months: 41.4%/48.3%/50.0%/53.4%. Although BSI incidence was higher in patients with a positive RS, it was marginally significant (*p* = 0.052). In 17/31(54%) of the BSIs in MDR-colonized patients, the causative agent was the same bacterium isolated on rectal swab. Moreover, the rate of MDR pattern in G-negative BSIs was higher in colonized patients than in non-colonized patients (61.9% vs. 17.4% *p* = 0.0003).

### 3.5 Mortality and outcome

**Figure 3:**
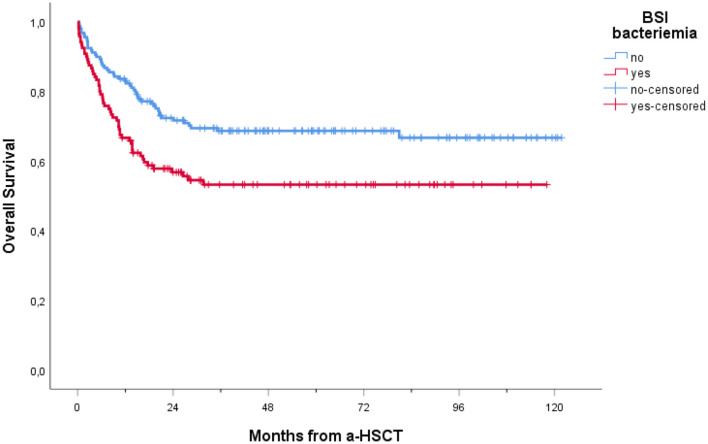
Overall survival of patients receiving a-HSCT stratified by BSI occurrence. Ticks on probability lines indicate dates of censoring at the last follow-up.

**Figure 4:**
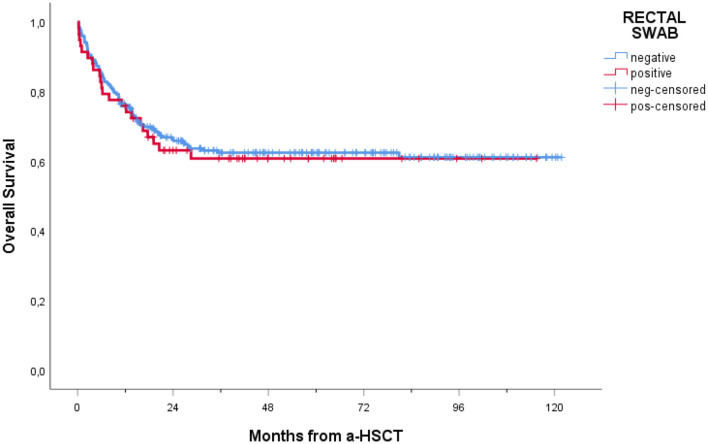
Overall survival of patients receiving a-HSCT stratified by colonization status. Ticks on probability lines indicate dates of censoring at the last follow-up.

In our cohort, 208 patients (74.6%) were alive at 1-year follow-up. One-year overall mortality was 25.4% (71 patients). The occurrence of BSI within 1 year post-transplantation had a significant impact on OS (*p* = 0.005), with a notably lower OS observed in patients experiencing BSI (Figure 3). Conversely, as shown in Figure 4, no consistent differences in OS were observed when comparing patients with positive RS with those with negative RS (*p* = 0.77).

**Figure 5:**
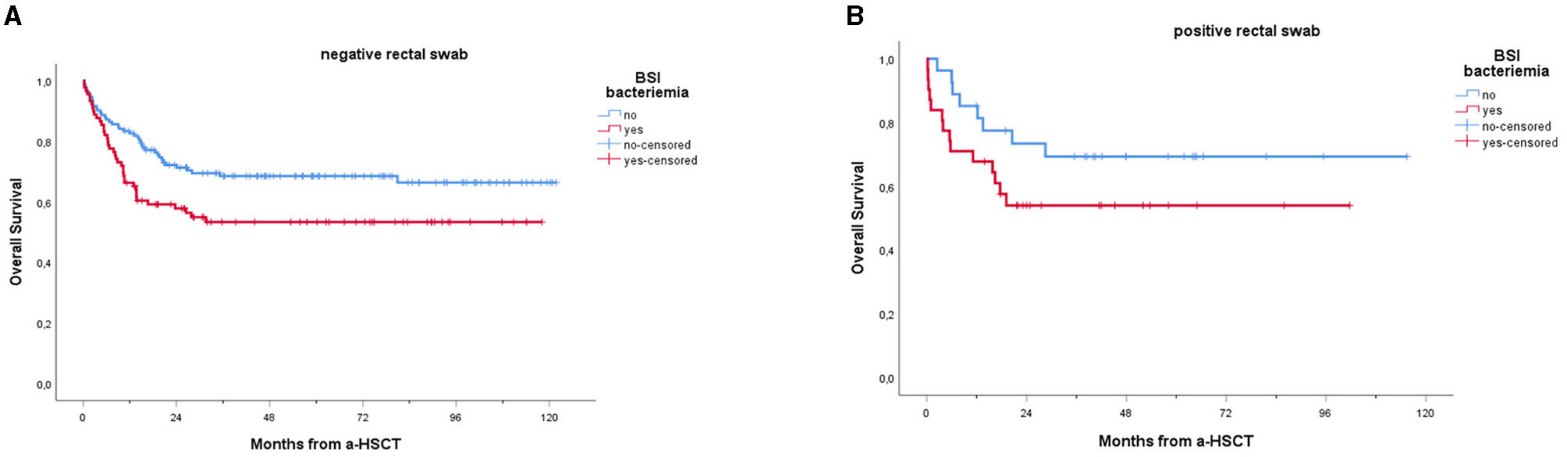
Overall survival of patients receiving a-HSCT stratified by BSI: a comparison of non-carriers **(A)** vs. carriers **(B)**. Ticks on probability lines indicate dates of censoring at the last follow-up. *A trend toward reduced OS can be observed among patients experiencing BSI, compared to non-BSI patients; this trend is confirmed for both carriers (A, p* = *0.018) and non-carriers (p* = *0.045), with no significant difference between the two groups*.

Furthermore, lower OS was recorded among patients with BSI vs. non-BSI patients, regardless of whether the RS was positive or negative (Figure 5).

Moreover, Table 5 shows univariate and multivariate Cox proportional hazard regression for OS after a-HSCT. At univariate analysis, factors significantly associated with OS were age at a-HSCT (HR 2.06, *p* = 0.016), gender (HR 0.51, *p* = 0.002), disease status at transplant (HR 2.34, *p* < 0.001), HCT-CI (HR 3.42, *p* < 0.001), stem cell source (HR 1.59, *p* = 0.050), conditioning regimen (HR 1.88, *p* = 0.006) and BSI (HR 2.31, *p* = 0.005). Multivariate analysis confirmed that male gender (HR 0.42, *p* < 0.001), advanced disease status at transplant (HR 1.99, *p* = 0.003), HCT-CI ≥ 3 (HR 3.02, *p* < 0.001), use of BM as stem cell source (HR 1.82, *p* = 0.018), and BSI (HR 1.52, *p* = 0.040) had an adverse significant impact on the OS of patients.

**Table 5:** Univariate and multivariate Cox proportional hazard regression for OS after a-HSCT.

	**Univariate models**		**Multivariate model**	
	**HR (95% CI)**	***p*** **-value**	**HR (95% CI)**	***p*** **-value**
**Age (years)**		0.049		0.513
41–60 vs. ≤ 40	1.50 (0.85–2.63)	0.160	1.13 (0.63–2.03)	0.672
61+ vs. ≤ 40	2.06 (1.14–3.70)	0.016	1.41 (0.75–2.64)	0.282
**Gender**				
Female vs. male	0.51 (0.34–0.77)	0.002	0.42 (0.27–0.65)	< 0.001
**Underlying disease**		0.953		
ALL vs. AML/MDS	0.93 (0.54–1.60)	0.783	–	–
Other vs. AML/MDS	0.95 (0.58–1.57)	0.842		
**Disease status at transplant**				
Advanced vs. early	2.34 (1.52–3.61)	< 0.001	1.99 (1.26–3.14)	0.003
**HCT–CI**		< 0.001		< 0.001
1–2 vs. 0	1.67 (0.95–2.95)	0.076	1.74 (0.98–3.09)	0.061
≥3 vs. 0	3.42 (1.96–5.96)	< 0.001	3.02 (1.71–5.33)	< 0.001
**Donor type**		0.378		
Haploidentical vs. MRD	1.41 (0.81–2.48)	0.227	–	–
MUD vs. MRD	1.04 (0.64–1.68)	0.875		
**HSC source**				
BM vs. PBSC	1.59 (1.00–2.53)	0.050	1.82 (1.11–2.98)	0.018
**Conditioning regimen**				
RIC vs. MAC	1.88 (1.20–2.96)	0.006	1.51 (0.95–2.42)	0.085
**ATG administration**				
Yes vs. no	0.83 (0.56–1.22)	0.337	–	–
**ATB prophylaxis**				
Yes vs. no	0.77 (0.50–1.19)	0.769	–	–
**aGVHD** ^*^				
Yes vs. no	0.69 (0.40–1.19)	0.181	–	–
**cGVHD** ^*^				
Yes vs. no	0.06 (0.02–0.19)	< 0.001	–	–
**BSI** ^*^				
Yes vs. no	2.31 (1.29–4.13)	0.005	1.52 (1.02–2.26)	0.040
**Rectal swab** ^*^				
Positive vs. negative	1.24 (0.62–2.48)	0.553	–	–

^*^Treated as time-dependent variable.

HR, hazard ratio; 95%CI, 95% confidence interval; AML, acute myeloid leukemia; MDS, myelodysplasitic syndrome; ALL, acute lymphoblastic leukemia; HCT-CI, hematopoietic cell transplantation comorbidity index;MRD, matched related donor; MUD, matched unrelated donor; HSC, haematopoieti stem cell; BM, bone marrow; PBSC, peripheric blood stem cell; RIC, reduced intensity conditioning; MAC, myeloablative conditioning; ATG, anti-thymocytes globulin; ATB prophylaxis, antibiotic prophylaxis; aGVHD, acute graft vs. host disease; cGVHD, chronic graft vs. host disease; BSI, blood stream infection.

## 4 Discussion

In this single-center study, we outlined the epidemiology of BSIs and clinical outcomes of 279 patients who underwent a-HSCT, focusing on the colonization of the rectal swabs by MDR pathogens. We also kept track of any BSIs that occurred within the 1st year after transplant, and identified risk factors that might have influenced infection and death.

In recent decades, the a-HSCT procedure has gained increasing approval and now represents a pivotal treatment option with an acceptable complication rate, even with HLA-haploidentical donors. Regrettably, infectious complications, particularly BSIs, still have a major impact on the survival of a-HSCT recipients, especially with the worldwide spread of MDR bacteria (De Rosa et al., 2017; Sorror et al., 2005).

In our cohort, 43% of patients developed BSIs in the post-transplant year, with median time onset of 10 days after a-HSCT. Likewise, previous works have reported similar average time onset, ranging between 4.5 and 15.5 days post-transplantation (Niyazi et al., 2023). During the observation period, we recorded an increasing trend in BSIs sustained by Gram-negative bacteria, which represented the main etiological agents. Previous studies have extensively described the role of Enterobacteriaceae and *P. aeruginosa* in BSIs occurring after a-HSCT (Macesic et al., 2014; Younes et al., 2017), representing a major issue for hematological patients. Nevertheless, Gram-positive bacteria are still a common cause of BSIs in hospitalized fragile patients and were involved in 50/120 (41.7%) BSIs in our study.

Patients undergoing HSCT are at high risk of intestinal mucosal damage due to intensive chemotherapy or radiation treatments. This explains the selection of MDR colonizers, particularly in cases of broad spectrum-antibiotic use, as they eliminate the beneficial bacteria in the gut that do not require oxygen, leading to intestinal dysbiosis (Chong and Koh, 2020). We identified a positive RS in 58 cases (20.8%). ESBL bacteria were the most common colonizers in our cohort, followed by CRE. (Forcina et al. 2018) described a comparable colonization rate, with ~17% of a-HSCT patients in their cohort exhibiting rectal colonization by MDR Gram-negative bacteria. Another Italian study reported that MDR Gram-negative bacteria colonized ~5% of HSCT recipients (Patriarca et al., 2017). Overall, carbapenem-resistant bacteria colonized 8.4% of our patients, a slightly lower percentage than the one described in a Turkish study on HSCT recipients (Demiraslan et al., 2017). The majority of positive RS were detected pre-transplant (55.2%). Nevertheless, the rate of colonization observed post-transplant remains significantly high (45%), underscoring the need for continued surveillance beyond the pre-transplant phase, rather than relying solely on pre-transplant screening to assess colonization risk.

According to the published data (Ferreira et al., 2018; Liss et al., 2012; Cao et al., 2022), our study confirmed high incidence of MDR-BSI was found among patients with MDR colonization. This finding aligns with a recent prospective multicenter cohort survey in Italy on hematological patients, revealing an independent association between MDR-positive RS and the occurrence of MDR Gram-negative bacteria BSIs (Trecarichi et al., 2023). It has also been shown that BSIs caused by CR-*K. pneumoniae* and *P. aeruginosa* have a worse outcome when compared to those caused by pathogens that are sensitive to carbapenems (Girmenia et al., 2017). Therefore, in this setting, we should prioritize interventions to reduce colonization, along with individualized, prompt, and active therapy for septic MDR-colonized patients. Researchers have recently developed a model that predicts the risk of a subsequent BSI in patients with hematologic malignancies who have CRE colonization (Liu et al., 2023).

In addition to rectal colonization, type of underlying disease, donor type, and disease status at the time of transplantation can also affect the risk of developing BSIs (Trecarichi et al., 2023; Forcina et al., 2018; Gill et al., 2023). Our analysis showed that patients with AML/MDS, those receiving transplants from MUD, and those in SD-PD had an increased risk of BSIs. This is likely due to the fact that these conditions are associated to a significant immune system impairment, increasing overall infectious risk. In particular, as pointed out by (Forcina et al. 2018), LAM/MDS is characterized by profound neutropenia and requires intensive conditioning regimens, which exacerbate the risk for microbial translocation and bloodstream infections. Additionally, the genetic difference between MUD donors and other types of donors increases the risk of GVHD, necessitating stronger immunosuppression and a longer period for immune reconstitution. Similarly, patients with SD-PD have higher infection risks compared to those in CR-PR due to severe immune function impairment secondary to previous aggressive treatments.

Furthermore, BSIs had a negative impact on OS in our cohort, adding to other better-known variables predicting poor outcomes in a-HSCT (e.g., disease not in remission at the time of transplant and GVHD). In line with previous research (Mikulska et al. 2009); (Stoma et al. 2016) and (Ustun et al. 2019) have recently confirmed, that BSIs negatively impact the OS of a-HSCT patients, regardless of whether the BSI occurs before or after engraftment. Also, Girmenia et al. found that GNB BSIs were a predictor of poor outcome in HSCT patients, being the main cause of death in 39.1% of a-HSCT recipients and 36.4% of auto-HSCT recipients who died before engraftment (Girmenia et al., 2017).

Interestingly, in our cohort, the negative impact of BSIs on OS seems to be independent of rectal colonization (refer to Figure 5). This aligns with a recent study by (Forcina et al. 2018), who found no difference in 14-day attributed mortality after MDR Gram-negative bacteria BSIs between non-carriers and carriers, despite the fact that in carriers, BSIs were mainly sustained by CR-*Klebsiella pneumoniae* or CR-PA, which are known to be associated with higher mortality (Girmenia et al., 2017). This might be related to the fact that in patients with known rectal colonization, the available microbiological data guide the choice and timing of antibiotic therapy, leading to the prompt initiation of targeted first-line therapy tailored to the RS colonizer.

In line with this, in our study, RS colonization did not have a significant impact on OS. The literature on the topic is controversial, and uniform evidence is currently lacking. (Heidenreich et al. 2017) found no clear and significant correlation between MDR colonization and an increase in mortality if prompt, effective antibiotic therapy is administered in cases of infective complications. In contrast, other research found that MDR bacteria rectal colonization lowers OS by raising the risk of transplant-related death and infectious complications after a-HSCT, even when antibiotics are used to target colonizing pathogens (Bilinski et al., 2016). For these reasons, considering MDR colonization as a relative contraindication to allogenic transplant is still a matter of debate (Patriarca et al., 2017).

Our study presents some limitations, such as those intrinsic in its retrospective, single-center nature, which limits the generalizability of our findings. Furthermore, the inclusion of patients undergoing multiple transplants may introduce selection bias, as these individuals present distinct clinical characteristics which could act as confounding factors. The decision was based on the clinical relevance of capturing the full spectrum of HSCT recipients encountered in real-world practice. In future analyses, we plan to explore the impact of transplant sequence more explicitly and to analyze these subgroups separately to further clarify their specific risk profiles. Additionally, in our center antibiotic prophylaxis as a preventive measure was withdrawn in 2016, midway through our observation period. The decision was driven by concerns about microbiota disruption, potentially increasing the risk of GVHD and antibiotic resistance. This change in internal protocol could have impacted local epidemiology, as suggested by a recent Italian perspective survey, reporting a favorable shift in the resistance patterns of Gram negative bacteria isolates following fluoroquinolone prophylaxis discontinuation (Trecarichi et al., 2023). We did not evaluate this effect in our study, potentially introducing a source of bias. Nevertheless, the cost-effectiveness of this strategy was analyzed on the same cohort in a separate study (Shbaklo et al., 2023).

Despite these limits, our study outlines that BSIs are still frequent complications in a-HSCT, with a significant impact on OS. This is especially true in the pre-engraftment period, when aplasia is still a major issue. Conversely, despite increasing the risk for BSI occurrence, MDR colonization itself is not associated with increased mortality. This finding may support the idea that MDR colonization is not *per se* a risk factor for mortality, although pre-transplant active surveillance, along with contact precautions and the early initiation of targeted combined therapy in carriers are crucial to minimize the risk of BSIs development and their associated adverse outcomes.

## Data Availability

The raw data supporting the conclusions of this article will be made available by the authors, without undue reservation.
